# Supplementary material for: Patterns of Intron Gain and Loss in Fungi
Source: PLoS Biol. 2004 Nov 30;2(12):e422. doi: 10.1371/journal.pbio.0020422 (PMC532390; doi:10.1371/journal.pbio.0020422)
Supplement: Table S1 — Also available at http://genes.mit.edu/NielsenEtAl/. (4.3 MB ZIP). [file pbio.0020422.st001.zip › NielsenEtAl/html/835.html]

AN1247.1.NCU07915.1.MG05062.1.FG03994.1


```
 CLUSTAL W (1.82) Multiple Sequence Alignments - Introns Inserted


Sequence 1: MG05062.1	276 aa
Sequence 2: FG03994.1	257 aa
Sequence 3: NCU07915.1	279 aa
Sequence 4: AN1247.1	252 aa
Alignment Length: 279 aa
Number Identitical Residues: 129 aa
Alignment Score (without introns) 6817


MG05062.1 	MTWELSRRRIIRTVNSKYIFDRV0PLLHFLIFLVEMAFVARLTSRFNAYYEERPI1LTMM
NCU07915.1	MSWDLTRRRWMRRLNSKYIFDRI0PLLHAIVFIIEMTIIARLTSRFNQFYNERPV1LTMM
FG03994.1 	MTWELSQRRFIRSINSKYIYGRI0PLLHTIVFLIEMAFIARLTARFNSYYDERPL1MTMM
AN1247.1  	-MFSTTRRRVLDGLNRRYIYGRL0PLLHTIIFMIEMAATARLAAKFNSYYAEKPV~LTTM
          	  :. ::** :  :* :**:.*: **** ::*::**:  ***:::** :* *:*: :* *

MG05062.1 	VTNA~VLGGIADTVAQTITAVKQRAARK--GPFHPNPKDDPIAIEIQELDRKNPLSDRDL
NCU07915.1	VTNA~VLAGVADTVAQSITAVRQRAVRKYPPGRGPNARDDFVAYEIHELDRKNPLNEQEL
FG03994.1 	VTNA~ILGGVADTVAQSITAIRERAIRQP-GGLK---KNDGVAIEIHELDRKNPFYERDL
AN1247.1  	VTNA0ILGGVADTVAQLITAFRARTGVRR--------GDDFIAIEIHDLEKEKPPAVGEL
          	**** :*.*:****** ***.: *:  :          :* :* **::*::::*    :*

MG05062.1 	IPDSRILPPPFDFERLTRFMAYGFCMAPVQFKWFKFLEKTFPITKTAAFGPAMKRVAMDQ
NCU07915.1	IPESRDLPPPFDFERLTRFMAFGFCMAPLQFKWFGFLERCFPITKKNAYQSALKRVAFDQ
FG03994.1 	IPDSVGLPPPFDFERLTRFMAYGFCMAPVQFKWFRFLERIFPVTKTSAFVPAMKRVAFDQ
AN1247.1  	G-HAKHTPPPFDFERLIRFMSYGFFMAPIQFQWFGFLSRTFPLAKKNPTFSALKRVACDQ
          	  .:   ********* ***::** ***:**:** **.: **::*. .  .*:**** **

MG05062.1 	LVFAPFG~IAAFFTVMTIAEGGGRRAVQNKLRDMYVPTLKANFAIWPAVQVINFRLMPVQ
NCU07915.1	LIFAPFG~LACFFTAMTLAEGGGKRGVYEKMRDLYVPTLKANYVLWPAVQVINFRLMPVS
FG03994.1 	LIFAPFG~LAVFYTTMTIAEGGGRRAVSNKLRDMYIPTLKANYVVWPAVQIVNFRLMPVQ
AN1247.1  	LLFAPFG1LVCFFSYMTIAEGGGKRALTRKFQDVYLPTLKANFVLWPAVQVLNFRVVPIQ
          	*:***** :. *:: **:*****:*.: .*::*:*:******:.:*****::***::*:.

MG05062.1 	FQL0PFVSTIGIAWTAYLSLSNAAEEAQP-TPHAYPDSPNLRLS
NCU07915.1	LQL0PFVSTVGIAWTAYLSLTNAAEDVQHTTPHRAPGSPDIRLS
FG03994.1 	FQL0PFVSTIGIAWTAYLSLTNSASE------------------
AN1247.1  	FQI0PFVSSVGIAWTAYLSLTNSAEEE-----------------
          	:*: ****::**********:*:*.:
```
